# Association Between the *miR-146a* Rs2910164 Polymorphism and Childhood Acute Lymphoblastic Leukemia Susceptibility in an Asian Population

**DOI:** 10.3389/fgene.2020.00886

**Published:** 2020-10-02

**Authors:** Dehua Zou, Jingwen Yin, Zhonglv Ye, Qiaoli Zeng, Chuan Tian, Yajun Wang, Qikang Chen, Riling Chen

**Affiliations:** ^1^Maternal and Child Research Institute, Shunde Women and Children's Hospital, Guangdong Medical University, Foshan, China; ^2^Department of Psychiatry, Affiliated Hospital of Guangdong Medical University, Zhanjiang, China; ^3^Department of Pediatrics, Affiliated Hospital of Guangdong Medical University, Zhanjiang, China; ^4^Clinical Research Center, Affiliated Hospital of Guangdong Medical University, Zhanjiang, China

**Keywords:** childhood acute lymphoblastic leukemia, *miR-146a*, rs2910164, Asian population, meta-analysis

## Abstract

**Background:** *miR-146a* has been demonstrated to be involved in normal hematopoiesis and the pathogenesis of many hematological malignancies by inhibiting the expression of its targets. Rs2910164(G>C) may modify the expression of the *miR-146a* gene, which might influence an individual's predisposition to childhood acute lymphoblastic leukemia (ALL). However, inconsistent findings have been reported on the association between the rs2910164(G>C) polymorphism and the risk of childhood ALL.

**Methods:** A comprehensive meta-analysis was performed to accurately estimate the association between the *miR-146a* rs2910164 polymorphism and childhood ALL among four different genetic models.

**Results:** This meta-analysis included Asian studies with a total of 1,543 patients and 1,816 controls. We observed a significant difference between patients and controls for the additive model (CC vs. GG: OR = 1.598, 95% CI: 1.003–2.545, *P* = 0.049) using a random effects model. Meanwhile, there was a trend of increased childhood ALL risk in the dominant model (CC + CG vs. GG: OR = 1.501, 95% CI: 0.976–2.307, *P* = 0.065), recessive model (CC vs. GG + CG: OR = 1.142, 95% CI: 0.946–1.380, *P* = 0.168) and allele model (C vs. G: OR = 1.217, 95% CI: 0.987–1.500, *P* = 0.066) between patients and controls.

**Conclusions:** Our findings suggest that the *miR-146a* rs2910164 CC genotype was significantly associated with childhood ALL susceptibility.

## Introduction

Acute lymphoblastic leukemia (ALL) is a very common malignancy among children, accounting for ~75% of leukemia cases among children (Pui et al., 2015). The incidence of this disease has continued to increase worldwide over the past several decades (Terracini, 2011). ALL is a clonal malignant disease that is characterized by an uncontrolled proliferation of immature cells, but its etiology remains unknown. MiRNAs are small non-coding RNAs that monitor gene expression post-transcriptionally. Previous studies have shown that miRNAs may play an important role in leukemogenesis (Schotte et al., 2010; Bottoni and Calin, 2013; Yan et al., 2013; Yin et al., 2014).

*miR-146a* has been identified as a modulator of cell differentiation and innate and adaptive immunity (Boldin et al., 2011; Rusca and Monticelli, 2011; Ghani et al., 2012; Labbaye and Testa, 2012). The abnormal expression of *miR-146a* is frequently observed in human diseases, such as inflammatory disorders and cancers (He et al., 2005; Iriyama et al., 2012; Petrovic et al., 2017; Shomali et al., 2017; Tan et al., 2018). Previous studies have reported that *miR-146a* is significantly increased in the peripheral blood samples of pediatric patients with ALL (Duyu et al., 2014; Yan et al., 2017), thus providing valuable insights into potential diagnostic or prognostic biomarkers. Lin showed that the absence of *miR-146a* can lead to leukemia in mice (Lin et al., 2015), which indicated that *miR-146a* might act as a leukemia suppressor. In addition, *miR-146a* might be involved in the progression of ALL by mediating the inflammatory response. Nuclear factor κB (NF-κB) signaling has been reported to be constitutively activated in a variety of leukemic cells. *miR-146a* could be upregulated by NF-κB (Justiniano et al., 2013; Taganov et al., 2015) and simultaneously inhibit the expression of TRAF6 and IRAK1, which are upstream regulatory proteins of NF-κB. Therefore, a negative feedback regulatory pathway is formed in the proliferation and differentiation of hematopoietic stem cells, similar to the development of hematological malignancies (Spierings et al., 2011; Zhao et al., 2011, 2013).

In the rs2910164 polymorphism, which is located in pre-*miR-146a*, the nucleotide substitution from G to C leads to a transformation from a G:U pair to a C:U mismatch in the stem structure of the *miR-146a* precursor and results in a reduced amount of mature *miR-146a* (Yue et al., 2011; Palmieri et al., 2014). Jazdaewski found that mature *miR-146a* with the C allele is less able to inhibit the target genes IRAK1 and TRAF6 than the G allele (Jazdzewski et al., 2008), which seems to be associated with the development of ALL.

A significant association of *miR-146a* rs2910164 with childhood ALL has been reported in an Iranian population (Hasani et al., 2014). This result was successfully replicated in two Chinese case-control studies (Liu et al., 2018; Pei et al., 2020). In contrast, three studies from Thailand, India and China failed to replicate the results (Chansing et al., 2016; Devanandan et al., 2019; Xue et al., 2019). Considering the conflicting results, whether *miR-146a* rs2910164 is associated with childhood ALL in Asian populations remains to be elucidated. In this study, we performed a meta-analysis to estimate the association of *miR-146a* rs2910164 with childhood ALL among four different genetic models in an Asian population.

## Materials and Methods

This study was pursuant to the Preferred Reporting Items for Systematic Reviews and Meta-analyses (PRISMA) guidelines. The PRISMA checklist is provided in Supplementary Table 1.

### Literature Search

The PubMed, Google Scholar, WanFang and Chinese National Knowledge Infrastructure databases were systematically searched for relevant studies using the words “*miR-146a* or *microRNA-146a*” “rs2910164” “leukemia” with no language or time restrictions. All studies were assessed by reading the title and abstract and irrelevant studies were excluded. Then, the full texts of the remaining studies were assessed to determine their eligibility.

### Inclusion and Exclusion Criteria

The study inclusion criteria were as follows: (a) case-control or cohort studies that assessed the association between the *miR-146a* rs2910164 polymorphism and the risk of childhood ALL; (b) studies in which all patients had been diagnosed with ALL by morphology, immunology, cytogenetic and molecular biology (MICM) in accordance with one of the following criteria: (i) bone marrow morphology standard: according to the 2016 WHO diagnostic criteria, the original and immature lymphocytes in bone marrow are not <20%, (ii) if the original and immature lymphocytes were <20%, a molecular diagnosis method was used to determine whether ALL pathogenic genes existed; (c) studies with data that could be used to estimate odds ratios (ORs) with corresponding 95% confidence intervals (CIs); and (d) studies published before April 30, 2020.

The exclusion criteria were as follows: (a) the study was not a case–control study; (b) the study was not related to acute lymphoblastic leukemia or 146a rs2910164; (c) the study lacked particular genotype data; (d) the genotype distribution of the control subjects was not in a Hardy–Weinberg equilibrium (HWE).

### Data Extraction

The following data were independently extracted from included studies and entered into a database to ensure the veracity of the data: first author's name, year of publication, population, genotyping techniques, number of patients and controls, genotype distribution, allele distribution, HWE and other information. Studies were excluded if they did not provide the above information.

### Statistical Analysis

The Hardy-Weinberg equilibrium was examined by Pearson's chi-squared test. Four genetic models were used in the study: the dominant model (CC +CG vs. GG), the recessive model (CC vs. GG + CG), the additive model (CC vs. GG) and the allele model (C vs. G). Genetic heterogeneity was evaluated using the Q-test and *I*^2^-test. *I*^2^ statistics range from 0 to 100%. Significant heterogeneity was defined as *P* < 0.01 and *I*^2^ > 50%. ORs with corresponding 95% CIs were calculated using the fixed effects model (Mantel-Haenszel) when no significant heterogeneity was observed; otherwise, a random effects model was used. The *Z*-test was used to test the significance of the ORs. To check the stability of our results, sensitivity analyses for the overall effect were conducted by excluding one study at a time. Additionally, Egger's and Begg's tests were used to assess publication bias. The statistical analyses were performed using the STATA v.16.0 software (Stata Corporation, Texas, USA), Review Manager 5.0.24 (The Nordic Cochrane Center, Denmark), R version 3.6.2 (R Core Team, Vienna, Austria) and RStudio version 1.2.1 (Certified B Corporations, Boston, USA).

## Results

### Study Inclusion and Characteristics

**Figure 1:**
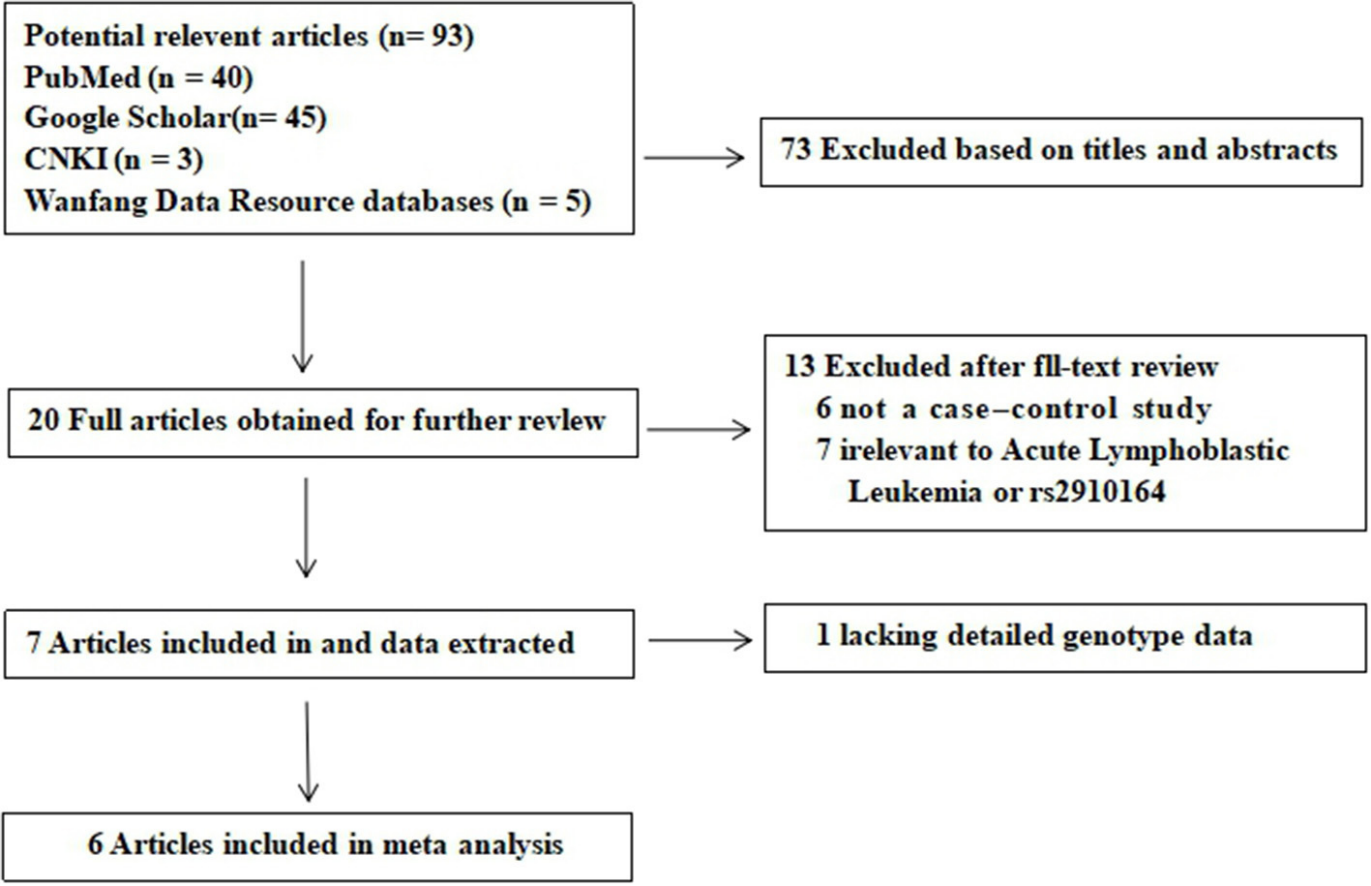
Flow diagram of the literature search and selection.

A total of 113 potential studies were retrieved through the initial search. Twenty duplicates were excluded. Then, the titles and abstracts of 93 studies were screened and 73 studies were excluded. The full texts of the remaining 20 articles were evaluated; 6 were excluded because they were not case-control studies and 7 were excluded because they were not related to acute lymphoblastic leukemia or rs2910164 and 1 study was excluded because it did not provide sufficient data. A flow chart of the study selection process is shown in Figure 1. There were 6 potentially relevant papers, including 5 written in English and 1 written in Chinese. A total of 1,543 childhood ALL patients and 1,816 healthy controls were included in the meta-analysis. Table 1 shows the characteristics of each study. Power analysis was conducted with the total sample size and revealed a power of 94.6% using an OR of 1.2 for the risk allele and a MAF of 0.39 for the C allele.

**Table 1:** Characteristics of the studies included in the meta-analysis.

**References**	**Population**	**Genotyping**	**Number of participants**		**Genotype distribution** ***(n)***			**Allele distribution** ***(n)***		**HWE (*P*)**	**MAF**
					**GG**	**GC**	**CC**	**G**	**C**		
Hasani et al. (2014)	Southeast Iranian	T-ARMS-PCR	75	ALL	7	46	22	60	90		0.400
			97	CTR	27	50	20	104	90	0.72	0.464
Chansing et al. (2016)	Thailand	PCR-RFLP	100	ALL	11	54	35	76	124		0.380
			200	CTR	31	96	73	158	242	0.95	0.395
Liu et al. (2018)	Baoding, China	PCR-RFLP	200	ALL	32	89	79	153	247		0.383
			100	CTR	29	41	30	99	101	0.07	0.495
Devanandan et al. (2019)	India	PCR-RFLP	71	ALL	27	32	12	86	56		0.394
			74	CTR	25	37	12	87	61	0.78	0.412
Xue et al. (2019)	Nanjing, China	SNaPshot	831	ALL	263	429	139	955	707		0.425
			1,079	CTR	369	541	169	1279	879	0.21	0.407
Pei et al. (2020)	Taiwan, China	PCR-RFLP	266	ALL	112	125	29	349	183		0.344
			266	CTR	90	117	59	297	235	0.08	0.442

*ALL, acute lymphoblastic leukemia; CTR, control; HWE, Hardy–Weinberg equilibrium; MAF, minor allele frequency*.

### Heterogeneity Analysis

The Cochran's Q test and the *I*^2^ statistics shown in Table 2 revealed that high heterogeneity among studies was detected in the CC +CG vs. GG (*I*^2^ = 74.9%), CC vs. GG + CG (*I*^2^ = 69.9%), CC vs. GG (*I*^2^ = 81.6%) and C vs. G (*I*^2^ = 79.5%) models for the rs2910164 polymorphism. As high heterogeneity was observed, sensitivity analysis was performed to analyze the sources of heterogeneity.

**Table 2:** Heterogeneity analysis with random-effect model.

**Genetic model**	***N***	**OR**	**95%-CI**	**Q**	**I^**2**^**
CC + CG vs. GG	6	1.2828	[0.8669; 1.8983]	19.93	74.90%
CC vs. GG + CG	6	0.9959	[0.6870; 1.4435]	16.61	69.90%
CC vs. GG	6	1.2635	[0.7010; 2.2776]	27.16	81.60%
C vs. G	6	1.0955	[0.8423; 1.4249]	24.37	79.50%

*OR, odds ratio; CI, confidence interval; I^2^, measure to quantify the degree of heterogeneity in meta-analyses*.

### Sensitivity Analysis

**Figure 2:**
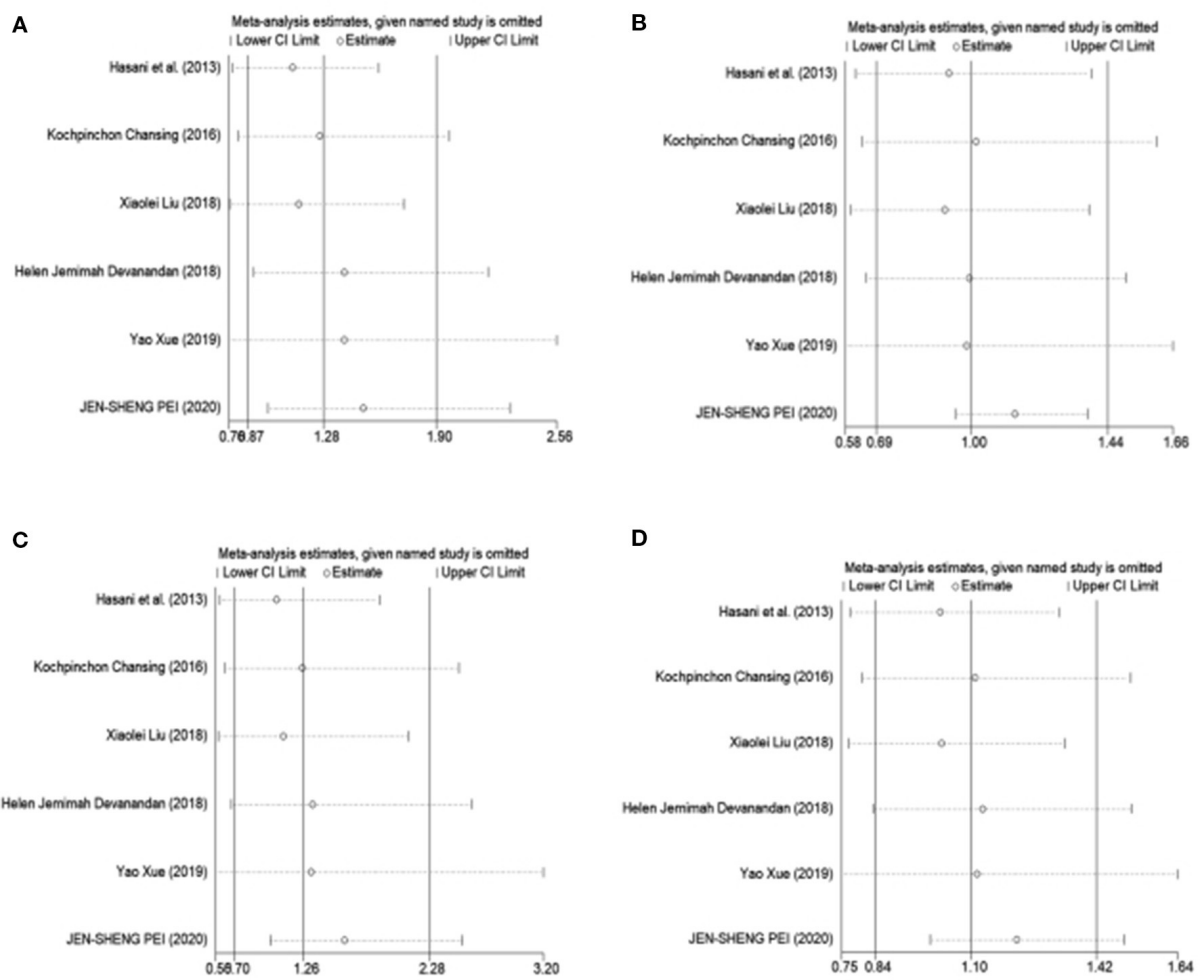
Sensitivity analysis was performed by removing one study at a time. **(A)** Dominant model, CC +CG vs. GG. **(B)** Recessive model, CC vs. GG + CG. **(C)** Additive model, CC vs. GG. **(D)** Allele model, C vs. G.

To estimate the influence of each study on the overall OR of the four genetic models and to analyze the sources of high heterogeneity, a sensitivity analysis was performed with a random effects model. The results are shown in Figure 2. Omitting Pei's study effectively reduced heterogeneity, especially in the recessive model (CC vs. GG + CG: *I*^2^ = 0.0%, *P* = 0.571). The other three models showed a moderate degree of heterogeneity (CC +CG vs. GG: *I*^2^ = 66.5%, *P* = 0.018; CC vs. GG: *I*^2^ = 57.9%, *P* = 0.050; C vs. G: *I*^2^ = 54.3%, *P* = 0.068, respectively) (Table 3).

**Table 3:** Heterogeneity analysis after omitting Pei's study with random-effect model.

**Genetic model**	**Study(*n*)**	**OR**	**95%-CI**	**Q**	**I^**2**^**
CC + CG vs. GG	5	1.5006	[0.9760; 2.3072]	11.94	66.50%
CC vs. GG + CG	5	1.1413	[0.9439; 1.3799]	2.92	0.00%
CC vs. GG	5	1.5977	[1.0027; 2.5455]	9.51	57.90%
C vs. G	5	1.2167	[0.9873; 1.4995]	8.74	54.30%

*OR, odds ratio; CI, confidence interval; I^2^, measure to quantify the degree of heterogeneity in meta-analyses*.

### Results of the Association Between *miR-146a* rs2910164 and Childhood ALL Meta-Analysis

**Figure 3:**
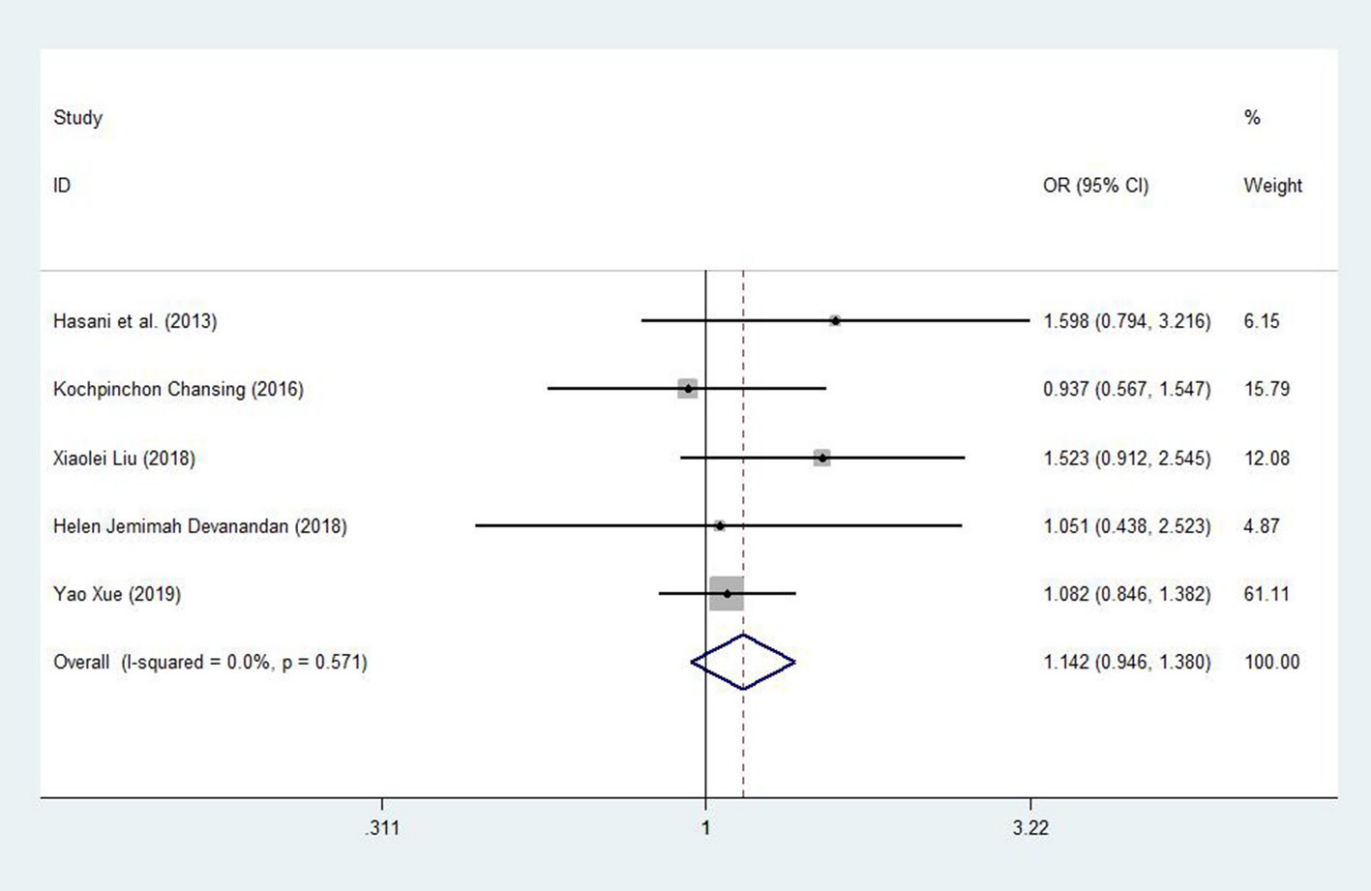
Meta-analysis with a fixed effects model for the association between the *miR-146a* rs2910164 polymorphism and childhood ALL susceptibility (recessive model, CC vs. GG + CG). OR, odds ratio; CI, confidence interval; I-squared, measure to quantify the degree of heterogeneity in meta-analyses.

**Figure 4:**
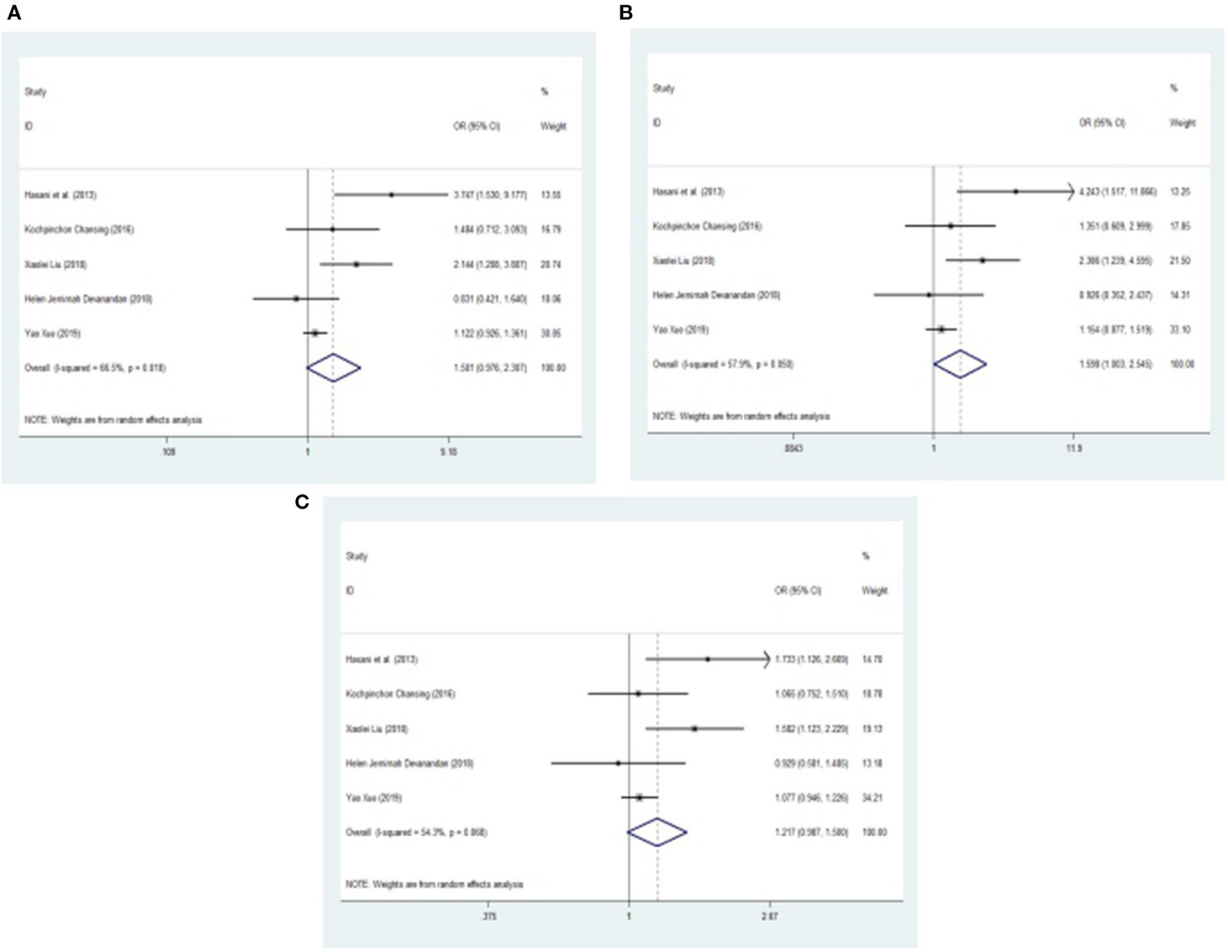
Meta-analysis with a random effects model for the association between the *miR-146a* rs2910164 polymorphism and childhood ALL susceptibility. **(A)** Dominant model, CC +CG vs. GG. **(B)** Additive model, CC vs. GG. **(C)** Allele model, C vs. G. OR, odds ratio; CI, confidence interval; I-squared, measure to quantify the degree of heterogeneity in meta-analyses.

After one study was omitted, there was a moderate degree of heterogeneity among studies. A fixed effects model was used to analyze the recessive model; the dominant, additive and allele models were analyzed with a random effects model. The results showed a significant difference between childhood ALL patients and controls for the additive model (CC vs. GG: OR = 1.598, 95% CI: 1.003–2.545, *P* = 0.049) and a trend of increased childhood ALL risk for the dominant model (CC +CG vs. GG: OR = 1.501, 95% CI: 0.976–2.307, *P* = 0.065), recessive model (CC vs. GG + CG: OR = 1.142, 95% CI: 0.946–1.380, *P* = 0.168) and allele model (C vs. G: OR = 1.217, 95% CI: 0.987–1.500, *P* = 0.066) between patients and controls (Figures 3, 4).

### Publication Bias

**Figure 5:**
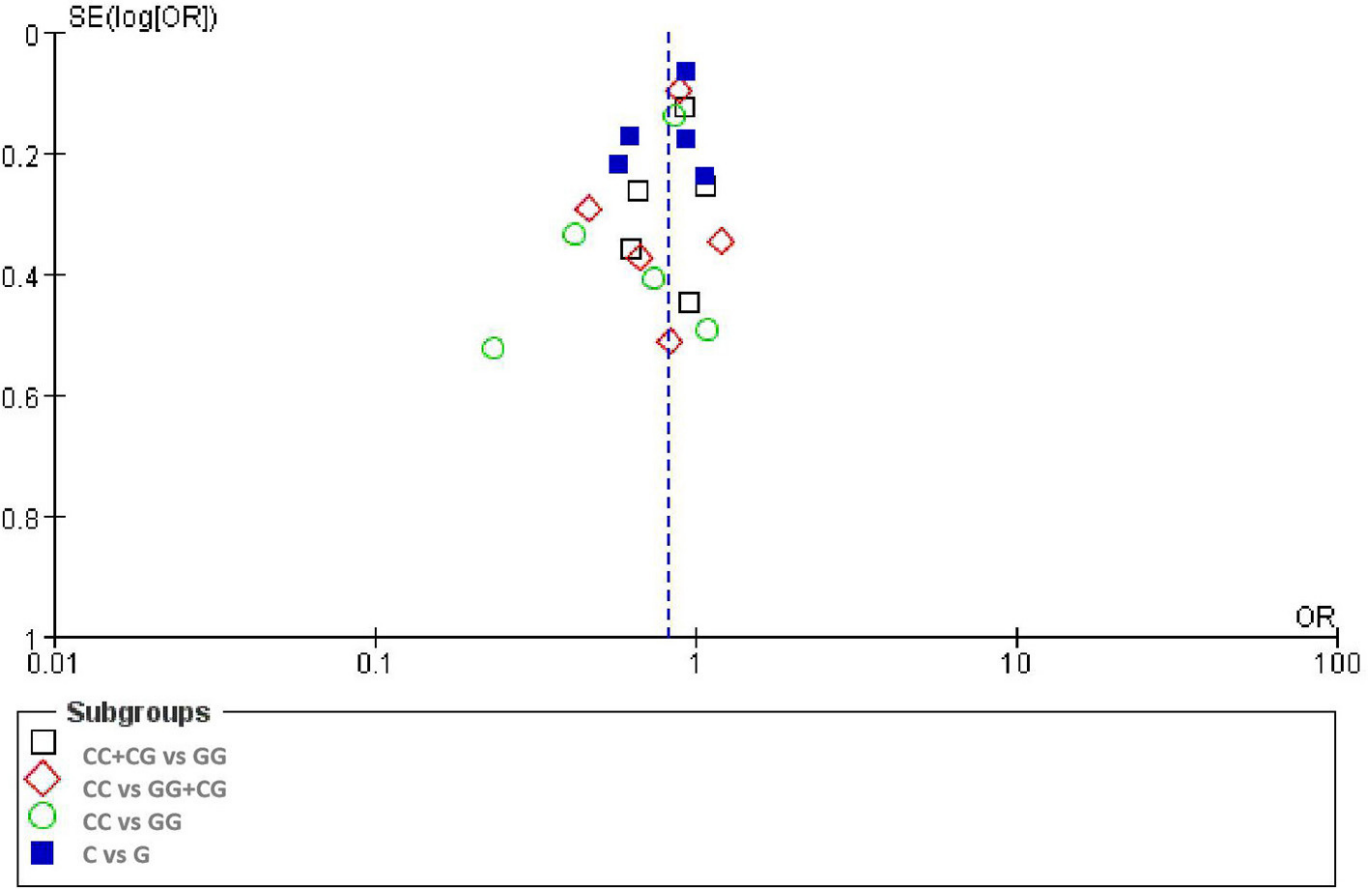
Funnel plot of the odds ratios in the meta-analysis.

There was no significant publication bias in any of the genetic models according to Begg's and Egger's tests (all *P* > 0.05, data not shown) and the funnel plot was symmetrical, as the studies did not coagulate into one quadrant of the funnel (Figure 5).

## Discussion

This meta-analysis included six Asian studies about the *miR-146a* rs2910164 loci and susceptibility to childhood ALL. Three studies reported an association between *miR-146a* rs2910164 and the risk of childhood ALL and the other three showed negative results. Moreover, the sample sizes of the individual studies were small, making it difficult to identify the possible small effect of rs2910164 on childhood ALL. Thus, this study enabled us to more accurately determine the association between rs2910164 and childhood ALL susceptibility due to the increased sample size and statistical power of the meta-analysis.

In this study, a total of 1,543 ALL patients and 1,816 controls were investigated to provide an overall evaluation of the association between the *miR-146a* rs2910164 polymorphism and childhood ALL. We conducted heterogeneity analysis and the results revealed that high heterogeneity among studies was detected in the four genetic models. Therefore, we explored the source of this heterogeneity via sensitivity analysis by omitting one study at a time. The results showed that omitting Pei's study could effectively reduce heterogeneity, especially in the recessive model and the other three models showed a moderate degree of heterogeneity. The most likely reasons for this heterogeneity might involve ethnicity, geographical region and the selection of control groups. The minor allele frequencies (MAFs) ranged from 0.395 to 0.495 in each study (Table 1). In Pei, Liu and Hasanis' studies, the MAF was higher than the NCBI SNP database in Asian populations. However, Pei's study sample sizes are larger than those of Liu and Hasanis. This is why Pei's study has a strong influence on heterogeneity. In addition, the populations of the control groups were not uniform. Individuals in the control group in Pei's study were determined to be cancer-free in accordance with the criteria set by the International Classification of Disease (ninth revision, defined by World Health Organization), but other studies did not explain the diagnostic criteria of the control groups. Thus, after omitting Pei's study, a fixed effects model was used to analyze the recessive model; the dominant, additive and allele models were analyzed with a random effects model. The results showed a significant difference between childhood ALL patients and controls for the additive model. Interestingly, there was a trend of increased childhood ALL risk in the other three modalities between patients and controls. These results showed that miR146a rs2910164 (G>C) was significantly associated with childhood ALL susceptibility.

In recent years, an increasing amount of data have demonstrated that *miR-146a* is related to normal hematopoiesis and the pathogenesis of some hematological malignancies by inhibiting the expression of its targets (Hua et al., 2011). The miR146a rs2910164 polymorphism has been extensively tested in different cancers. The rs2910164 CG or GG genotype was linked to a significantly decreased risk for lung cancer compared to the CC genotype (Jeon et al., 2014). In addition, the rs2910164 CC genotype may be devoted to breast cancer susceptibility in Europeans (Lian et al., 2012). For childhood ALL. Previous studies found that the rs2910164 CC or CG genotype significantly increased the risk of ALL (Hasani et al., 2014; Liu et al., 2018). The miR146a rs2910164 GG genotype was significantly related to a decreased susceptibility to childhood ALL (Pei et al., 2020). This study confirms that the miR146a rs2910164 polymorphism (G>C) contributes to childhood ALL susceptibility among Asians.

There are still some limitations in our study. Firstly, due to the limited examination of *miR-146a* rs2910164 in childhood ALL, only six studies were included in the meta-analysis. Secondly, the current research only includes Asian studies and there is an urgent need to conduct research using large samples of other ethnic groups across the world. Thirdly, the complexity of ALL, which is the result of the interaction of genetic and environmental factors, may affect the results. Among individuals with the same genotype, their susceptibility to ALL may be different due to the geographical environment lifestyle and other factors of the diverse population (Garzon et al., 2010).

In conclusion, our work contributed important evidence regarding the association between the *miR-146a* rs2910164 CC genotype and susceptibility to childhood ALL in an Asian population. Given the relatively small sample size of this study, more large-sample studies including different ethnic populations are needed to validate these results.

## Data Availability Statement

The datasets presented in this study can be found in online repositories. The names of the repository/repositories and accession number(s) can be found in the article/Supplementary Material.

## Author Contributions

DZ, JY, ZY, and QZ were responsible for the statistical analysis, study design, and manuscript preparation. DZ and CT managed the literature searches and analyses. This study was supervised by YW, QC, and RC. All authors contributed to the article and approved the submitted version.

## Conflict of Interest

The authors declare that the research was conducted in the absence of any commercial or financial relationships that could be construed as a potential conflict of interest.
